# Acellular Pertussis Vaccine Inhibits *Bordetella pertussis* Clearance from the Nasal Mucosa of Mice

**DOI:** 10.3390/vaccines8040695

**Published:** 2020-11-19

**Authors:** Jana Holubová, Ondřej Staněk, Ludmila Brázdilová, Jiří Mašín, Ladislav Bumba, Andrew R. Gorringe, Frances Alexander, Peter Šebo

**Affiliations:** 1 Institute of Microbiology of the Czech Academy of Sciences, Videnska 1083, 142 20 Prague 4, Czech Republic; hejnova@biomed.cas.cz (J.H.); stanek@biomed.cas.cz (O.S.); ludmila.brazdilova@biomed.cas.cz (L.B.); masin@biomed.cas.cz (J.M.); bumba@biomed.cas.cz (L.B.); 2 Public Health England, Research and Development Institute, Porton Down, Salisbury SP4 0JG, UK; andrew.gorringe@phe.gov.uk (A.R.G.); Frances.Alexander@phe.gov.uk (F.A.)

**Keywords:** *Bordetella pertussis*, whooping cough, vaccines, nasal colonization

## Abstract

*Bordetella pertussis* whole-cell vaccines (wP) caused a spectacular drop of global pertussis incidence, but since the replacement of wP with acellular pertussis vaccines (aP), pertussis has resurged in developed countries within 7 to 12 years of the change from wP to aP. In the mouse infection model, we examined whether addition of further protective antigens into the aP vaccine, such as type 2 and type 3 fimbriae (FIM2/3) with outer membrane lipooligosaccharide (LOS) and/or of the adenylate cyclase toxoid (dACT), which elicits antibodies neutralizing the CyaA toxin, could enhance the capacity of the aP vaccine to prevent colonization of the nasal mucosa by *B. pertussis*. The addition of the toxoid and of the opsonizing antibody-inducing agglutinogens modestly enhanced the already high capacity of intraperitoneally-administered aP vaccine to elicit sterilizing immunity, protecting mouse lungs from *B. pertussis* infection. At the same time, irrespective of FIM2/3 with LOS and dACT addition, the aP vaccination ablated the natural capacity of BALB/c mice to clear *B. pertussis* infection from the nasal cavity. While wP or sham-vaccinated animals cleared the nasal infection with similar kinetics within 7 weeks, administration of the aP vaccine promoted persistent colonization of mouse nasal mucosa by *B. pertussis.*

## 1. Introduction

The Gram-negative coccobacillus *Bordetella pertussis* is the major agent of a highly contagious respiratory infectious disease called whooping cough [1]. Pertussis pneumonia elicited by *B. pertussis* and accompanied by hyperleukocytosis and other systemic effects of the pertussis toxin used to be the prime cause of infant mortality in developed countries in the pre-vaccine era. Introduction of effective pertussis vaccines containing killed whole *B. pertussis* cells (wP), formulated into a diphtheria–tetanus–whole-cell pertussis (DTwP) combination vaccine with the diphtheria and tetanus toxoids, enabled a spectacular reduction of clinical pertussis incidence since the 1950s [2]. Nevertheless, pertussis remains the least-controlled vaccine-preventable infectious disease that was estimated to have accounted for over 24 million of whooping cough cases and more than 160,000 deaths due to pertussis world-wide in 2014 [3]. Moreover, in the past two decades, the highly effective but reactogenic wP vaccines have been progressively replaced in the most developed countries by less reactogenic acellular pertussis (aP) vaccines [2]. These comprise 1 to 5 purified antigens and are typically administered in a pediatric hexavaccine combination. Clinical evidence accumulated over the past decade shows that aP vaccines efficiently prevent infant mortality, but confer a substantially less complete and shorter lasting protection from whooping cough, which is further reduced by subsequent Tdap (tetanus toxoid plus a reduced dose of the diphtheria and pertussis vaccine) booster immunizations [4,5,6,7,8,9,10]. Consequently, pertussis resurged and whooping cough outbreaks have reappeared in the most developed countries within 7 to 12 years from completion of the change from wP to aP vaccines. This phenomenon is currently potentiated by the aP vaccine-driven emergence of *B. pertussis* strains that do not produce pertactin (PRN), the key antigenic target of opsonizing antibodies elicited by the aP vaccines [11,12,13,14,15]. While increased surveillance and more accurate diagnostic technologies also contributed to increased reporting of pertussis [16,17], transmission of *B. pertussis* by asymptomatic and/or non-diagnosed aP-vaccinated carriers emerges as a major factor of pertussis resurgence. A growing body of evidence indicates that the aP vaccines fail to prevent nasopharynx infection and *B. pertussis* transmission in fully vaccinated populations [18,19].

Early seminal work of Mills and Redhead (1993) uncovered the involvement of Th1-polarized CD4^+^ T cells in elimination of *B. pertussis* infection and pointed to the difference of Th1 polarization of immune responses elicited by the wP vaccine or experimental infection, as compared to predominant Th2 polarization of immune responses triggered by the aP vaccine in mice [20,21]. Further work revealed that natural infection and the wP vaccine, but not the aP vaccine, induce *B. pertussis*-specific IL-17 and IFN-γ-secreting CD103^+^ CD44^+^ CD69^+^ CD4^+^ tissue resident memory T cells (T_RM_) that appear to play a key role in early protection of the airway mucosa from *B. pertussis* infection [19,22,23,24,25,26,27,28].

wP and aP vaccines also differ substantially in the breadth of antibodies induced, and wP contains innate memory-activating TLR ligands [29,30]. The wP vaccine delivers many bacterial surface antigens, including type 2 and type 3 fimbriae (FIM2/3) and lipooligosaccharide (LOS), both targeted by protective agglutinating and opsonizing serum antibody responses that represent the only as yet clinically established correlate of protection from pertussis illness in humans [31]. In contrast, the protection provided by aP vaccines against critical pertussis bronchopneumonia mainly relies on induction of serum antibodies that neutralize pertussis toxin (PT) and prevent its systemic effects, such as the life-threatening hyperleukocytosis that contributes to refractory pulmonary hypertension and heart failure [32]. Depending on composition, aP vaccines also induce serum IgG antibodies against *B. pertussis* adhesins, such as PRN, filamentous hemagglutinin (FHA), or FIM2/3.

For historical reasons, the other key immunosuppressive toxin of *B. pertussis,* the adenylate cyclase toxin-hemolysin (adenylate cyclase toxin (ACT), AC-Hly or CyaA), has not been targeted by licensed aP vaccine formulations. The protective antigen potential of the toxoid of ACT (dACT) contaminated by *Escherichia coli* LPS (lipopolysaccharide) has previously been established in BALB/c mice [33,34]. dACT was also protective when used as LPS-free antigen [35], and its capacity to enhance the protective efficacy of a 1/8 diluted aP vaccine (Infanrix^®^) in the model of mouse lung infection by *B. pertussis* was demonstrated in NIH mice [36]. Moreover, under quite different experimental conditions, theRTX (repeats in toxin) fragment of ACT was also found to enhance the protective efficacy of 1/80 diluted (Infanrix^®^) vaccine in CD-1 mice [37]. Given that ACT ablates the sentinel bactericidal functions of host neutrophils and macrophage cells [38,39,40], we reasoned that induction of ACT-neutralizing antibodies by a dACT-supplemented aP vaccine might enable the aP vaccine to elicit protection against nasal colonization by *B. pertussis*. The results reported here confirm that inclusion of dACT into a FIM2/3 and LOS-supplemented diluted aP vaccine modestly enhances its already high capacity to elicit sterilizing immunity in mouse lungs and protect them from *B. pertussis* infection. However, dACT and FIM2/3 with LOS addition did not override the aP vaccine-induced inhibition of clearance of *B. pertussis* infection from the nasal cavity of BALB/c mice.

## 2. Materials and Methods

### 2.1. B. pertussis Strains and Growth Conditions

The Tohama I-derived *B. pertussis* strain BPSM (Str^R^) was a kind gift of C. Locht (Institut Pasteur de Lille, Lille, France). The bacteria were grown on Bordet-Gengou (BG) agar plates (Difco, Franklin Lakes, NJ, USA) supplemented with 1% glycerol, 15% defibrinated sheep blood (LabMediaServis, Jaromer, Czech Republic) and 100 µg/mL streptomycin at 37 °C in a 5% CO_2_ atmosphere for 72 h to visualize hemolysis. Liquid cultures were obtained by growing bacteria overnight in modified Stainer–Scholte medium (SSM) [41] supplemented with 3 g/L of Casamino Acids (Difco, Franklin Lakes, NJ, USA) and 1 g/L of heptakis (2.6-di-O-dimethyl) β-cyclodextrin (Sigma-Aldrich, St. Louis, MO, USA) to a mid-exponential phase (*B. pertussis* OD_600_ = 1.0) at 37 °C. Bacterial suspensions were diluted in SSM to the density required for intranasal inoculation. To check for viable colony forming units (CFUs) in the inocula, serially diluted suspensions were plated on BG agar containing streptomycin (100 µg/mL).

The mScarlet fluorescent protein [42] was expressed in *B. pertussis* Tohama I from the pBBR1MCS [43] derived plasmid carrying the *mScarlet* gene under the control of the *B. pertussis* fha promoter.

### 2.2. Antigens

The native PT and PRN antigens were a kind gift of Dr. Umesh Shaligram from Serum Institute of India Pvt. Ltd. (SIIPL, Pune, India). Native FIM2/3 and FHA and recombinant dACT (188GS189) were purified as described previously [44,45,46]. Native PRN for addition into the diluted Hexacima^®^ vaccine was obtained from NIBSC (Hertfordshire, UK, cat. # 18/154), and native PRN for ELISA was from SIIPL.

Recombinant 6xHis-tagged rPRN for use in splenocyte restimulation was produced in *E. coli* BL21 cells and purified from urea extracts of inclusion bodies by Ni-NTA agarose chromatography including an endotoxin removal step by washing the resin with bound PRN with 1% of Triton X100 [45]. Purified rPRN was dialyzed against 8 M urea in 50 mM Tris pH 8, and the homogeneity of the protein was analyzed by SDS-PAGE. The endotoxin content was determined with a Limulus amebocyte lysate assay (QCL-1000, Lonza, Walkersville, MD, USA).

### 2.3. Composition of Vaccines Used in the Study

All vaccines used in this study were formulated into 200 µL doses with the antigen content described in Table 1 and were admixed no longer than 1 h before the administration. The commercial aP-containing pediatric hexavaccines (D/T/aP/Hib/HepB/IPV) used in this study were Hexacima^®^ (Sanofi Pasteur S.A., Lyon, France) and Infanrix^®^ (GlaxoSmithKline Biologicals S.A., London, UK). The wP-containing pediatric pentavaccine (D/T/wP/Hib/HepB) used was Pentavac^®^ SD (Serum Institute of India PVT. LTD.). Aliquots of the commercial vaccines were diluted in phosphate-buffered saline (PBS) with alum adjuvant at a final concentration of 0.208% Al(OH)_3_ (*w*/*v*), so that 1/160 human dose (HD) (Hexacima^®^) aP, or 1/20 HD (Hexacima^®^ or Infanrix^®^) aP, or 1/4 HD (Pentavac^®^ SD) wP was contained in 200 µL. The 200 µL doses of the supplemented aP vaccines further contained the indicated amounts 1, 3, or 5 μg/dose of added fimbriae 2/3 (FIM2/3) and of associated LOS (6000, 18,000 and 30,000 endotoxin units (EU), respectively), and/or a 0.2 μg/dose of added PRN (into 1/20 HD of Hexacima^®^ only) and/or 30 μg/dose of dACT (Table 1). The unvaccinated control mice received 200 µL of PBS with 0.208% Al(OH)_3_ (*w*/*v*).

### 2.4. Ethics Statement

All animal experiments were approved by the Animal Welfare Committee of the Institute of Molecular Genetics of the Czech Academy of Sciences, v. v. i., in Prague, Czech Republic. Handling of animals was performed according to the *Guidelines for the Care and Use of Laboratory Animals*, the Act of the Czech National Assembly, Collection of Laws no. 246/1992. Permission no. 37/2018 was issued by the Animal Welfare Committee of the Institute of Molecular Genetics of the Czech Academy of Sciences in Prague.

### 2.5. Mouse Immunization and Intranasal Infection

Female BALB/cByJ mice (Charles River, Écully, France) were vaccinated by intraperitoneal injection at 5 weeks of age with the vaccines described in Table 1 and were boosted with the same vaccine 14 days later. Control mice received PBS with alum (0.208% Al(OH)_3_ (*w*/*v*)). Three weeks later, six mice from each group were withdrawn for serological analysis and for splenocyte isolation and restimulation (see below). Remaining immunized mice were anesthetized by intraperitoneal (i.p.) injection of ketamine (80 mg/kg) and xylazine (8 mg/kg) in 0.9% saline and challenged intranasally by the indicated bacterial dose applied in two aliquots of 10 µL per nare of the mouse. Three to six mice per group were anesthetized as described above at the indicated time points (e.g., at 2 h, 7, 14, 27, 35 and 50 days post challenge). For ethical reasons (3R requirements), three mice per group were used in the ex-post control of the inoculated dose (the first time point of 2 h) and at time points where no or very low CFU counts were expected based on preliminary data. Groups of 5 or 6 mice were sacrificed and analyzed at the other time points for comparisons between individual vaccine groups and to the control mice group. Lungs and nasal cavities with turbinates were aseptically removed and homogenized in physiological saline with tissue grinders (Heidolph mechanical stirrers, Model RZR 2020, Merck, Darmstadt, Germany) and tissue homogenizer (IKA Ultra turrax T25, Sigma-Aldrich, St. Louis, MO, USA), respectively. Nasal homogenates were cleared by centrifugation for 10 min at 900 rpm (217× *g*), and dilutions of lung and nasal homogenates were plated on BG agar supplemented with 15% defibrinated sheep blood and 100 µg/mL of streptomycin. CFU were counted after incubation for 4 days at 37 °C.

### 2.6. Analysis of Antibody Responses

Blood was collected from anesthetized animals by retro-orbital puncture. Serum was separated by centrifugation at 5000× *g* for 10 min at 8 °C and stored at −80 °C until use. Antibodies to *B. pertussis* antigens were determined by ELISA as described by van der Ark et al. [47]. Briefly, wells of ThermoFisher Nunc Maxisorp 96-well plates were coated overnight with 0.5 µg of the respective antigens in 100 μL of 100 mM carbonate buffer pH 9.6 for PT, FHA, PRN, and FIM2/3 and in 50 mM Tris pH 8 for dACT. For the anti-ACT ELISA, the coating and the washing buffer (50 mM Tris buffer pH 8, pH 7.4, respectively) were supplemented with 2 mM CaCl_2_ to facilitate the folding of the immunodominant RTX domain of the ACT protein.

For determination of total IgG by whole bacterial cell ELISA, plates were coated overnight at 37 °C with 100 μL of *B. pertussis* cells diluted to OD_600_ of 0.025 in PBS. The wells were blocked with 200 μL of PBS with 1% BSA. Ten-fold serial dilutions of sera (100 μL) were added starting at 1:10 dilution for control mouse sera and 1:100 for vaccinated mouse sera. Secondary antibodies labelled with horseradish peroxidase were used (100 μL). Upon reaction of the added OPD and H_2_O_2_, A_492_ was read. For titration of total IgG, sheep anti-mouse antibody (GE, Chicago, IL, USA, clone NA931V) was used at dilution 1:3000. For titration of IgG1 isotype, goat anti-mouse antibody (Invitrogen, Carlsbad, CA, USA, cat. n. A10551, polyclonal) was used at dilution of 1:1000. For titration of IgG2a isotype, goat anti-mouse antibody (Abcam, Cambridge, UK, cat. n. ab97245, polyclonal) was used at dilution of 1:50,000. Antibody titers were calculated from the inflection points of the titration curves.

### 2.7. Opsonophagocytic Uptake

The opsonophagocytic uptake assay (OPA) was adapted from Fabbrini et al. [48] using HL-60 cells. Briefly, HL-60 cells were differentiated for 5 days in 60 mL volumes of Roswell Park Memorial Institute medium (RPMI 1640; Sigma-Aldrich, St. Louis, MO, USA) supplemented with 10% (*v/v*) FBS and 1.25% (*v/v*) DMSO, at an initial cell concentration of 4 × 10^5^ mL. Cell differentiation status was verified by flow cytometry using a FITC-labeled anti-CD11b antibody (OKM1 clone). The cells were washed in Hank’s Balanced Salt Solution (HBSS)with 1% BSA and 0.1% glucose but devoid of Ca^2+^ and Mg^2+^ and resuspended in HBSS with Ca^2+^ and Mg^2+^ plus 1% BSA and 0.1% glucose.

Serum samples were split in two aliquots, and in one aliquot, the complement was heat-inactivated for 30 min at 56 °C. Both heat-inactivated and untreated sera were used in the opsonophagocytic assays for comparison.

*B. pertussis* expressing the mScarlet fluorescent protein was grown overnight in Steiner–Scholte medium at 37 °C. The bacteria (5 × 10^6^ cells/well) were opsonized in a total volume of 50 μL of complete HBSS with 1% BSA and 0.1% glucose upon addition of 5 μL of tested sera (10%) for 30 min at 37 °C with agitation. A total of 10^5^ differentiated HL-60 cells in 50 μL were added to each well, diluting the serum to 5%. Cells were incubated with bacteria for 30 min at 37 °C with agitation. Uptake was stopped by the addition of 100 μL of ice-cold PBS, and the cells were washed, stained with FITC-labeled anti-CD11b (OKM1 clone) antibody, washed twice, resuspended in 100 μL of ice-cold PBS and kept on ice until analyzed by flow cytometry. Only CD11b^high^ cells were included in the analysis by the gating strategy. Mean fluorescence intensity (MFI) of the ingested bacteria was measured. Data from individual experiments were normalized to the control (opsonization with naïve serum of control mice).

### 2.8. Agglutination Titer Determination

Agglutination titer of the sera was measured using the agglutination reagents (TestLine Clinical Diagnostics, Brno, CZ), according to the manufacturer‘s instructions. Briefly, a two-fold dilution series of each serum in physiological saline solution in U-bottom wells was set up, and equal volumes of the agglutinogen (standardized inactivated *B. pertussis* suspension) were added. The starting dilution was 1:4, and the final dilution was 1:8192. A serum with known agglutinating titer from the same manufacturer was used as a positive control, and physiological saline solution was used as a negative control. The samples were incubated for 2.5 h in 37 °C humidified atmosphere and further incubated overnight (18 h) at 20 °C. A homogenous agglutinate marked a positive reaction, while sediment at the bottom of the well marked a negative reaction. The titer was determined as the last dilution yielding a homogenous agglutinate. The agglutination assays were repeated three times.

### 2.9. Splenocyte Restimulation and Secreted Cytokine Determination

Gently disrupted spleens were homogenized by passage through a strainer. Splenocyte suspensions were depleted of erythrocytes by treatment with ammonium chloride-potassium, washed twice and cultured in RPMI 1640 medium with L-glutamine supplemented with 10% FCS, 1% penicillin and 1% streptomycin at 3 × 10^5^ cells/well in 96-well flat-bottomed tissue culture plates. Spleen cells from mice immunized with the wP vaccine were cultured (stimulated) in a total volume of 200 µL per well with 3 × 10^6^ heat-killed *B. pertussis* BPSM cells (MOI 1:10). Spleen cells from mice immunized with the aP vaccine were stimulated in a total volume of 200 µL per well with the combinations of antigens (e.g., urea denatured PT, native FHA, rPRN, native FIM2/3 and dACT) in combinations reflecting the aP vaccine compositions used, 2 µg/mL of detoxified pertussis toxin (dPT), FHA or FIM2/3, 5 µg/mL of rPRN and 1 µg/mL of dACT, respectively. PBS and phorbol myristate acetate (PMA)/ionomycin (eBiosciences, Sigma-Aldrich, St. Louis, MO, USA) served as negative and positive controls, respectively. Splenocyte cultures were incubated at 37 °C in 5% CO_2_ atmosphere at 90% humidity for 48 and 72 h. Cell viability was checked by Trypan blue exclusion before and after the incubation.

Cytokine concentrations in splenocyte culture supernatants were determined using a custom built ProcartaPlex cytokine bead assay (ThermoFisher Scientific, Waltham, MA, USA) on a Bio-Plex 200 instrument (Bio-Rad, Hercules, CA, USA) according to the manufacturer’s instructions. The instrument was calibrated on assay days using a Bio-Plex Calibration kit (Bio-Rad, #171,203,060), and data were validated using a Bio-Plex Validation kit (Bio-Rad, #171,203,001) within two weeks of each assay.

### 2.10. ACT Cytotoxicity Neutralization Assay

THP-1 cells were cultured at 37 °C in a humidified air/CO_2_ atmosphere in RPMI medium supplemented with 10% heat-inactivated FCS, penicillin (100 i.u./mL), streptomycin (100 µg/mL) and amphotericin B (250 ng/mL). Prior to assays, RPMI was replaced with Dulbecco’s Modified Eagle’s Medium (D-MEM) (1.9 mM Ca^2+^) without FCS.

For neutralization of ACT binding to THP-1 cells, intact ACT (1 µg/mL) was preincubated in D-MEM medium (without FCS) for 15 min on ice in the presence of the 1:50 diluted mouse serum before THP-1 cells (10^6^) were added and the mixture was incubated for an additional 30 min at 4 °C. Unbound ACT toxin was removed by three washes in D-MEM, and upon transfer to a fresh tube, the cells were lyzed with 0.1% Triton X-100 for determination of the cell-bound AC enzyme activity. ACT binding in the presence of naïve serum was taken as 100%. *n* = 4 biological replicates performed in duplicates.

For neutralization of the cytotoxic activity, ACT (125–1000 ng/mL) was incubated with serum samples (dilution 1:50) for 15 min at room temperature in D-MEM medium, and the samples were added to the THP-1 monocyte/macrophage cells (1.5 × 10^5^). Cells were incubated at 37 °C for 2 h in a humidified air/5% CO_2_ atmosphere, and the number of viable cells was determined using the Cell proliferation reagent kit WST-1 (Roche Applied Science, Penzberg, Germany), based on the conversion of the tetrazolium salt WST-1 (4-[3-(4-Iodophenyl)-2-(4-nitro-phenyl)-2H-5-tetrazolio]-1,3-benzene sulfonate) to formazan product (A_450 nm_). The viability of control cells (buffer treated only) was taken as 100%. *n* = 2 biological replicates performed in duplicates.

### 2.11. Statistical Analysis

Statistical analysis was carried out using the algorithms incorporated in the GraphPad Prism 7 package. Two-way analysis of variance (ANOVA) followed by Tukey’s multiple comparison test was used to analyze the statistical significance between groups. *p* values of less than 0.05 were considered statistically significant. * (*p* < 0.05); ** (*p* < 0.01); *** (*p* < 0.001); **** (*p* < 0.0001).

## 3. Results

### 3.1. Limiting Dose of aP Supplemented with dACT and FIM2/3 Plus LOS Elicits Partial Protective Immunity against B. pertussis Infection of Mouse Lungs But No Protection of the Nasal Cavity

**Figure 1:**
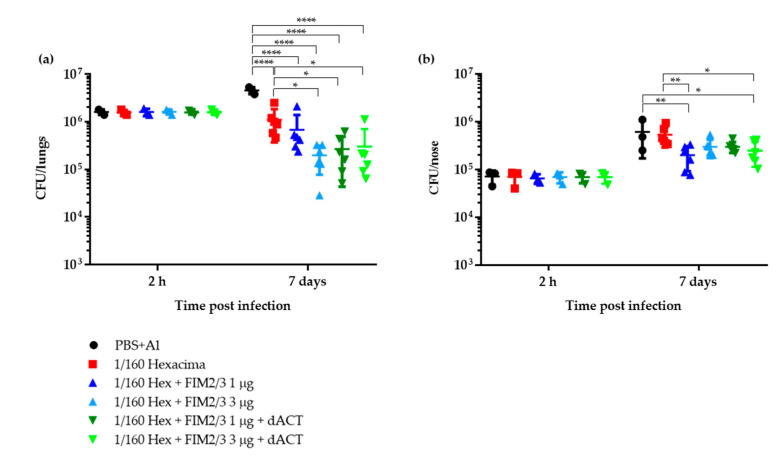
Protection against intranasal *B. pertussis* challenge in BALB/c mice immunized by 1/160 human dose (HD) of antigen-supplemented Hexacima^®^ aP vaccine. Mice were immunized intraperitoneally on days 0 and 14 with 1/160 HD of the aP vaccines (1/160 HD Hexacima^®^, 1/160 HD Hexacima^®^ + FIM2/3 (1 µg), 1/160 HD Hexacima^®^ + FIM2/3 (3 µg), 1/160 HD Hexacima^®^ + FIM2/3 (1 µg) + dACT (30 µg) and 1/160 HD Hexacima^®^ + FIM2/3 (3 µg) + dACT (30 µg)). Mice injected with phosphate-buffered saline (PBS) + alum (0.208% *w*/*v*) served as negative controls. Mice were challenged intranasally with *B. pertussis* BPSM (1.4 × 10^6^ CFU in 2 × 10 µL) on day 35. Mice were sampled at day 0 + 2 h and day 7 post challenge for enumeration of bacteria in the lungs (**a**) and in the nasal cavity (**b**). Results represent the bacterial counts for three to six mice per group ± SD. Two-way ANOVA followed by Tukey’s multiple comparison test was used to analyze the statistical significance between groups. Only significant differences are indicated. * (*p* < 0.05); ** (*p* < 0.01); **** (*p* < 0.0001).

We first sought to define the fraction of a human dose (HD) of the aP vaccine that would allow detection of an added protective activity of the dACT toxoid, and/or of the added FIM2/3 and LOS antigens, against colonization of the nasal mucosa and of mouse lungs. Several challenge doses delivered in various volumes were tested in preliminary experiments, and the dose of 10^6^ *B. pertussis* BPSM (Str^R^, Tohama I-derived) CFU delivered in 2 × 10 μL into the nares of mice was chosen to achieve a high enough CFU number retained in the nasal cavity. This inoculum enabled reliable CFU counting in homogenates of the nasal tissue, as well reliable colonization tracking in the lungs, where a clear increase in CFU was observed in the control animals within the first 7 days of infection (Figure 1a). Preliminary titration of the vaccine dose (Figure S1) indicated that a 1/160 HD, containing 0.156 μg dPT and 0.156 μg FHA in PBS with alum (Al(OH)_3_ 0.208% *w*/*v*) and injected intraperitoneally twice at two week intervals, conferred a still statistically significant, but already sufficiently low level of protection against mouse lung infection by intranasal application of 10^6^ CFU of *B. pertussis* BPSM. As shown in Figure 1a, at this aP vaccine dose, there was only about a five-fold difference in the CFU counts recovered on day 7 after infection from lungs of sham-vaccinated mice that received PBS with alum and mice vaccinated twice with 1/160 HD of Hexacima^®^ aP vaccine diluted in PBS with alum. Addition of 1 or 3 μg of FIM2/3 antigen containing 6000 or 18,000 EU of *B. pertussis* LOS per dose of such vaccine, respectively, improved the protection slightly, yielding about five-fold reduction of bacterial load in infected lungs compared to 1/160 HD of the aP vaccine only (ten-fold compared to control). However, the difference was not enhanced any further upon addition of 30 μg of the LPS-free dACT toxoid per vaccine dose. Moreover, as shown in Figure 1b, the 1/160 HD of aP did not on its own confer any protection against bacterial colonization of the mouse nose. Only about a two-fold decrease in bacterial burden in the nose was observed when the diluted aP was supplemented with FIM2/3 and LOS antigens alone, or in combination with dACT. In these experiments, the 1/160 HD of aP supplemented with dACT alone was not tested, as anti-ACT antibodies are proposed to improve efficacy only when opsonizing antibodies are induced and no such antigen is contained in the PT + FHA-containing aP (Hexacima^®^) vaccine.

### 3.2. 1/20 Human Dose of dPT + FHA aP Vaccine Supplemented with PRN and FIM2/3 Induces Sterilizing Immunity in Mouse Lungs But Inhibits Elimination of B. pertussis Infection from the Nasal Cavity

**Figure 2:**
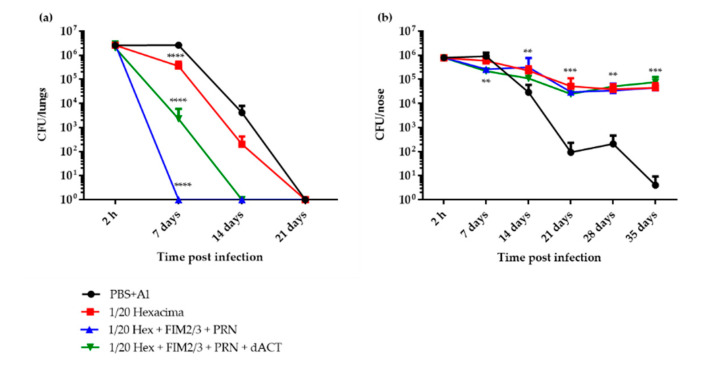
Protection against intranasal *B. pertussis* challenge in BALB/c mice immunized by 1/20 HD of antigen-supplemented Hexacima^®^ aP vaccine. Mice were immunized intraperitoneally on days 0 and 14 with 1/20 HD of aP vaccines (1/20 HD Hexacima^®^, 1/20 HD Hexacima^®^ + FIM2/3 (5 µg), 1/20 HD Hexacima^®^ + FIM2/3 (5 µg) + PRN (0.2 µg) and 1/20 HD Hexacima^®^ + FIM2/3 (5 µg) + PRN (0.2 µg) + dACT (30 µg). Mice injected with PBS + alum (0.208% *w*/*v*) served as negative controls. Mice were challenged intranasally with *B. pertussis* BPSM (3.4 × 10^6^ CFU in 2 × 10 µL) on day 35 and sampled at indicated time points (2 h, 7, 14, 21, 28 and 35 days) post challenge for enumeration of bacteria in the lungs (**a**) and in the nasal cavity (**b**). Results represent the means of bacterial counts for three to six mice per group ± SD, as defined in Materials and Methods. Two-way ANOVA followed by Tukey’s multiple comparison test was used to analyze the statistical significance between groups. Only significant differences vs. negative control mice are indicated. ** (*p* < 0.01); *** (*p* < 0.001); **** (*p* < 0.0001).

The concentration of the aP vaccine was raised to 1/20 HD (e.g., 1.25 μg dPT and 1.25 μg FHA per dose) and a limiting amount (0.2 μg per dose) of the potent opsonizing antibody-inducing antigen PRN was added. We supposed that neutralization of the phagocyte activity-ablating action of ACT by the dACT-induced neutralizing antibodies (Figure S2a) would observably potentiate the impact of the presence of opsonizing anti-PRN antibodies. As indeed shown in Figure 2a, compared to the PBS with alum control, two doses of the 1/20 HD of aP vaccine alone (Hexacima^®^ diluted in PBS with alum) conferred only a modest protection of mouse lungs against a challenge with 3.4 × 10^6^ CFU of BPSM bacteria delivered into mouse nares in 2 × 10 μL. However, the use of such a modified vaccine did not enable bacterial clearance to occur much faster than in control animals and took 21 days to complete. In contrast, supplementation of the 1/20 HD of the aP vaccine with 5 μg per dose of FIM2/3 and 30,000 EU of LOS and with 0.2 μg PRN per vaccine dose brought about a significant enhancement of the protective immunity and yielded complete clearance of *B. pertussis* from mouse lungs within 7 days after infection. Intriguingly, the potency of the FIM2/3 and PRN-supplemented 1/20 HD aP vaccine was rather compromised by further addition of 30 μg per dose of LPS-free dACT. Even more intriguingly, neither 1/20 HD of the aP, nor its antigen-supplemented versions, conferred any protection against persistent colonization of the mouse nose (Figure 2b). Compared to sham immunization with PBS with alum (0.208 *w*/*v*), only about ten-fold lower bacterial burden was found on day 7 after infection in the noses of mice that received 1/20 HD of aP supplemented with FIM2/3 (5 μg), LOS (30,000 EU) and PRN (0.2 μg), with or without the addition of 30 μg of dACT. Moreover, all mice that received any of the aP vaccine combinations maintained a steady level of nasal colonization at around 10^5^ CFU per nose for at least 35 days after infection. In contrast, the sham-immunized control mice that received PBS with alum essentially cleared the nasal infection by that time.

### 3.3. 1/20 Human Dose of a dPT + FHA + PRN aP Vaccine Supplemented with FIM2/3 and dACT Induces Sterilizing Immunity in Mouse Lungs But Blocks Elimination of B. pertussis Infection from the Nasal Cavity

**Figure 3:**
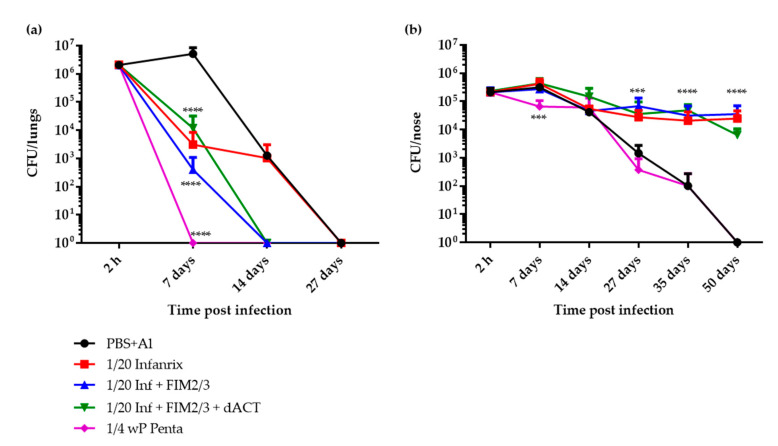
Protection against intranasal *B. pertussis* challenge in BALB/c mice immunized by 1/20 HD of antigen-supplemented Infanrix^®^ aP vaccine. Mice were immunized intraperitoneally on days 0 and 14 with 1/4 HD of wP and 1/20 HD of aP vaccines (1/20 HD Infanrix^®^, 1/20 HD Infanrix^®^ + FIM2/3 (5 µg) and 1/20 HD Infanrix^®^ + FIM2/3 (5 µg) + dACT (30 µg). Mice injected with PBS + alum (0.208% *w*/*v*) served as negative controls. Mice were challenged intranasally with *B. pertussis* BPSM (2.7 × 10^6^ CFU in 20 µL) on day 35 and sampled at indicated time points (2 h, 7, 14, 27, 35 and 50 days) post challenge for enumeration of bacteria in the lungs (**a**) and in the nasal cavity (**b**). Results represent the means of bacterial counts for three to five mice per group ± SD, as defined in Materials and Methods. Two-way ANOVA followed by Tukey´s multiple comparison test was used to analyze the statistical significance between groups. Only significant differences vs. negative control mice are indicated. *** (*p* < 0.001); **** (*p* < 0.0001).

The intriguing inhibition of nasal clearance of *B. pertussis* infection in mice that received 1/20 HD of the aP vaccine prompted us to reproduce the result with another diluted commercial hexavaccine that contains the same amounts of dPT and FHA but also contains 8 μg of PRN per human dose (Infanrix^®^). As shown in Figure 3a, a near identical result was obtained. The 1/20 HD of the aP vaccine alone, containing 0.4 μg of PRN per dose, conferred immunity, allowing for a reduction in the bacterial burden in the lungs 7 days post infection that was significant when compared with PBS with alum control, but still on day 14, a comparable bacterial burden in the lungs as in sham-vaccinated animals was observed. The protection conferred by the 1/20 HD of aP was importantly enhanced upon addition of 5 μg of FIM2/3 and 30,000 EU of LOS per dose and was again slightly lower upon further addition of 30 μg of dACT. Both mice groups that received the supplemented aP vaccine cleared the infection from the lungs by day 14 post infection. For comparison, a group of mice immunized with 1/4 HD of a pediatric wP-based pentavaccine (Pentavac^®^ SD) was also used, and the wP-vaccinated group of mice cleared the lung infection within 7 days. Remarkably, despite the rather high rate of lung infection clearance, all aP-immunized mice—irrespective of the addition of FIM2/3 with LOS and/or dACT, and despite the presence of 0.4 μg of PRN per vaccine dose and of ACT-neutralizing antibodies (Figure S2b)—again maintained a steady level of *B. pertussis* colonization at 10^5^ CFU per nose for 50 days post infection (Figure 3b).

### 3.4. The Supplemented aP Vaccine Induces a Th_2_-Polarized Immune Response with IgG1 Isotype-Agglutinating Antibodies That Enhance Opsonophagocytic Uptake of B. pertussis Bacteria In Vitro

**Figure 4:**
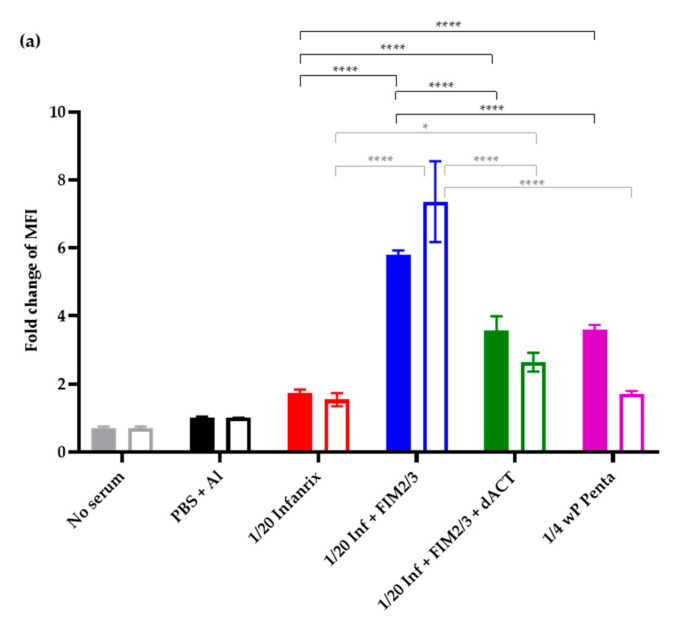

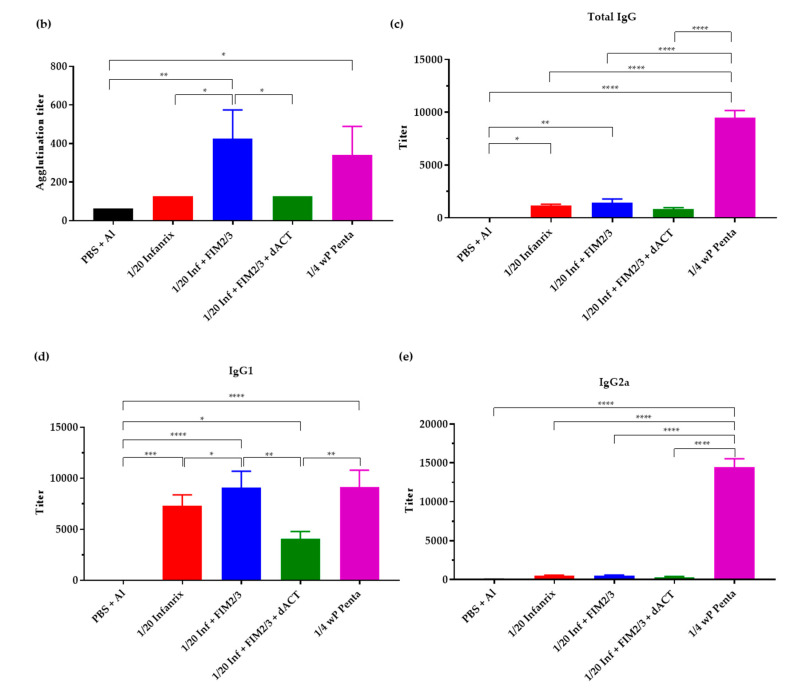
Innate and adaptive immune response elicited in BALB/c mice immunized by 1/20 HD of antigen-supplemented Infanrix^®^ aP vaccine. (**a**) Sera from mice immunized as described in the legend of Figure 3 were obtained on day 35 and heat-treated for complement inactivation (empty bars) or not (full bars) before use for opsonization of mScarlet-expressing *B. pertussis* bacteria. Opsonophagocytic uptake into differentiated HL-60 cells was performed as described in the Materials and Methods and analyzed by flow cytometry. A representative experiment performed in triplicate is shown. The fold change of mean fluorescence intensity (MFI) was calculated by dividing the MFI obtained in the presence of immune plasma by the MFI obtained upon incubating cells with bacteria in the presence of plasma from PBS + alum vaccinated mice. Data are shown as mean ± SD. Tukey´s multiple comparison test was used to analyze the statistical significance between groups. Only significant differences are indicated. * (*p* < 0.05); **** (*p* < 0.0001). (**b**) Agglutination titers of the sera were determined as the last dilution that agglutinated the standardized *B. pertussis* cell reagent (TestLine Clinical Diagnostics). *n*=3 biological replicates performed in triplicates. Data shown as mean ± SD. Tukey´s multiple comparison test was used to analyze the statistical significance between groups. Only significant differences are indicated. * (*p* < 0.05); ** (*p* < 0.01). (**c**–**e**) Antibody responses to *B. pertussis* elicited by the various vaccine combinations were determined by whole-cell ELISA on plates coated by heat-killed *B. pertussis* BPSM using anti-total mouse IgG or IgG1 or IgG2a-specific secondary antibodies. Results represent the mean antibody titers determined as the inflection points of the titration curves ± SD. A pool of sera of six mice per group was used in triplicates, and two-way ANOVA followed by Tukey´s multiple comparison test was performed to analyze the statistical significance between groups. * (*p* < 0.05); ** (*p* < 0.01); *** (*p* < 0.001); **** (*p* < 0.0001).

Intriguingly, the failure to clear the infection from the nose was not due to a failure to trigger the expected immune responses. The added antigens elicited production of antibodies that increased opsonophagocytic uptake of mScarlett-expressing *B. pertussis* BPSM bacteria by differentiated HL-60 cells, as shown in Figure 4a. Moreover, the 1/20 HD of aP (Infanrix^®^) supplemented with 5 μg of FIM2/3 and 30,000 EU of LOS per dose triggered formation of *B. pertussis* bacteria-agglutinating serum antibodies at levels as high as the 1/4 HD of a pediatric wP-based pentavaccine (Figure 4b). Hence, a functional antibody response to FIM2/3 and LOS was induced (see also Figure S3). However, the 1/20 HD of aP and the antigen-supplemented 1/20 HD aP induced much lower titers of total IgG against whole *B. pertussis* cells than the vaccination with 1/4 HD of the wP vaccine (Figure 4c). Furthermore, the antibody responses elicited by the aP vaccine compositions were strongly polarized towards the IgG1 antibody isotype (Figure 4d), and very low titers of IgG2a isotype antibodies recognizing whole *B. pertussis* cells were induced by the 1/20 HD of aP vaccines, whether supplemented or not (Figure 4e).

**Figure 5:**
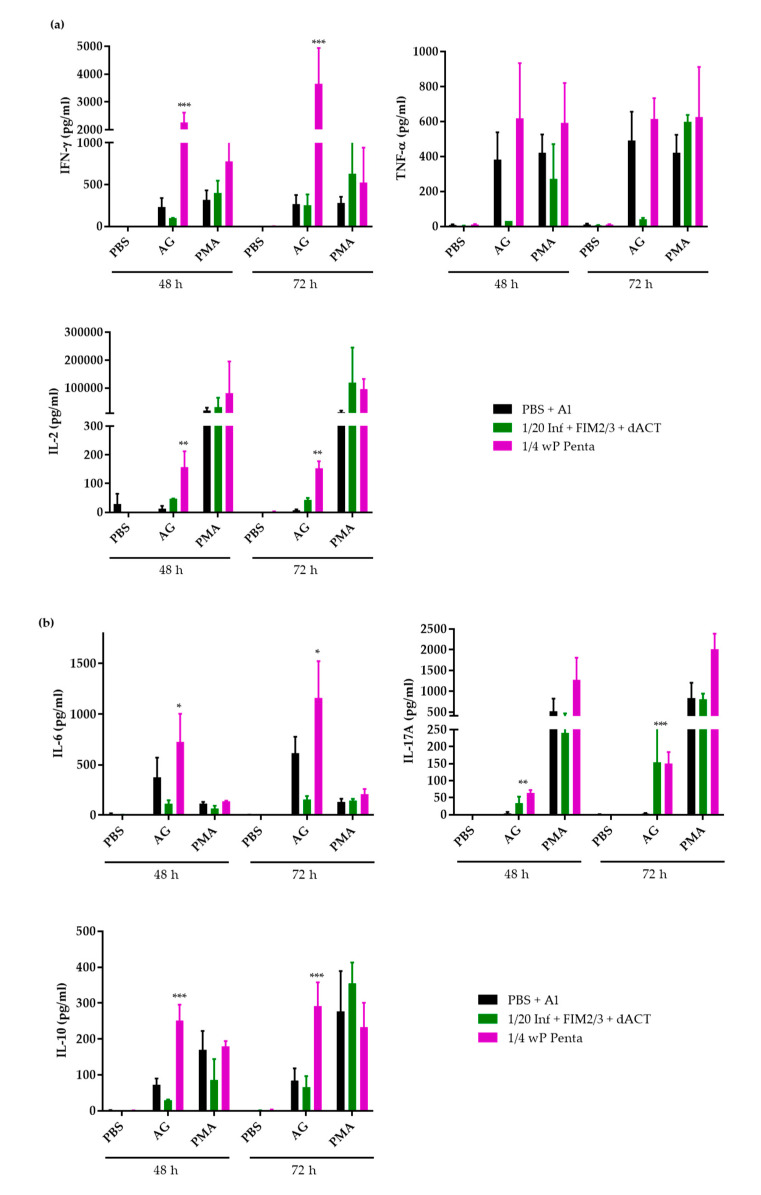
Cytokine secretion by restimulated splenocytes of BALB/c mice immunized by 1/20 HD of antigen-supplemented Infanrix^®^ aP vaccine. (**a**) Th1 cytokines. (**b**) IL-6, Il-17A and IL-10 cytokines. Splenocytes from four mice per time point were restimulated for 48 or 72 h using the antigen combinations used for immunizations (AG = mixture of all antigens used for immunizations). Two supernatants per time point were pooled and cytokine concentrations were measured in duplicates using a cytokine bead assay, as described in detail in the Materials and Methods. Sham stimulation with PBS and unspecific polyclonal stimulation with phorbol myristate acetate (PMA) plus ionomycin were used as negative and positive control, respectively. Data shown as mean ± SD. * (*p* < 0.05); ** (*p* < 0.01); *** (*p* < 0.001) vs. negative control mice.

Indeed, compared to splenocytes of wP-vaccinated mice, upon stimulation with the *B. pertussis* antigens for 48 and 72 h, the total splenocytes of mice immunized with the 1/20 HD of aP supplemented with FIM2/3, LOS and dACT produced significantly less of the Th1 cytokine IFN-γ and of TNF-α, as well as of the IL-2 cytokines (Figure 5a). Importantly, the IFN-γ and TNF-α responses were substantially lower in the aP-vaccinated mice, despite addition of FIM2/3 with LOS, than in sham-vaccinated mice that received only PBS with alum. The same was true also for the IL-6 and IL-10 cytokines, while the IL-17A cytokine secretion by splenocytes of aP-vaccinated mice was increased over that of control mouse splenocytes and, after 72 h of antigenic stimulation, approached IL-17A levels produced by splenocytes from wP-vaccinated mice (Figure 5b).

## 4. Discussion

We report that various parenterally applied acellular pertussis vaccine compositions triggered a sterilizing immunity in mouse lungs but compromised the natural capacity of BALB/c mice to clear the *B. pertussis* infection from the nasal cavity. Vaccination with 1/20 HD of aP vaccine, whether supplemented with FIM2/3, LOS and dACT antigens or not, ablated the capacity of BALB/c mice to clear the infection from the nasal mucosa, despite enabling a rapid and full clearance of *B. pertussis* from infected lungs. The mechanism responsible for this troubling observation remains to be established, but the hypothesis of mispriming or compromising of mucosal immune defenses by the aP vaccine appears rather plausible.

Fractions of the human dose of aP vaccines were found to efficiently elicit immunity protecting mouse lungs from *B. pertussis* infection [21,23,28,36,37,49,50,51,52]. However, the comparison of published data is difficult. Various mouse strains, immunization routes, vaccine doses and compositions were used in various regimens, and varying infectious challenge doses of different *B. pertussis* strains were applied in varying suspension volumes, either directly into mouse nares, or as inhaled aerosol. Moreover, the two aP vaccines formulated into pediatric hexavaccines available in Europe differ in composition, with one (Hexacima^®^, Sanofi) containing 25 μg of detoxified PT (dPT) plus 25 μg of filamentous hemagglutinin (FHA) and the other (Infanrix^®^, GSK) also containing 8 μg of PRN per dose.

In this study we used the BALB/c mouse lung infection clearance model, since the BALB/c mice are prone to development of Th2-polarized immune responses, like human infants are, and Th polarization has been shown to play a major role in immune protection against *B. pertussis* infections [21,51,53]. Indeed, Dubois and co-workers (C. Locht, personal communication) recently found that immunization by the aP vaccine blunted the expansion of *B. pertussis* antigen-specific IL-17 and IFN-γ-secreting CD103^+^ CD44^+^ CD69^+^ CD4^+^ tissue resident memory T cells (T_RM_) in the nasal mucosa of *B. pertussis*-infected BALB/c mice (Dubois et al., -submitted). Such T_RM_ responses were found to be required for an initial protection of mouse airway mucosa from *B. pertussis* infection in the more Th1-prone C57Bl/6 mice and were only induced by natural infection, or by the wP vaccine, but not by the conventional alum-adjuvanted aP vaccines [22,23,25,27,28]. These data thus call for investigation of the mechanism by which by the alum-adjuvanted aP vaccine would blunt the establishment of the mucosa-homing T_RM_ cells.

Intriguingly, in our experiments, despite a high efficacy in the lung clearance model and the induction of Th1/Th17-polarized immune responses, even immunization with 1/4 HD of the wP vaccine conferred only about a 10-fold reduction of the initial bacterial burden in the noses of immunized mice on day 7 (c.f. Figure 3b). Vaccination with the wP did not accelerate the clearance of nasal infection when compared with the natural capacity of sham-vaccinated BALB/c mice to eliminate *B. pertussis* from the nasal cavity. Both control and wP-immunized mice cleared the nasal infection with similar kinetics within 50 days after challenge. In contrast, Wilk and colleagues observed a substantial wP-induced protection of C57Bl/6 mouse nasal mucosa from *B. pertussis* infection and colonization [23,27,28]. This discrepancy could be due to difference of immune polarization of responses in the C57Bl/6 *versus* BALB/c mice and the different bacterial doses used for nose infection. In this study, we have directly applied over 10^6^ CFU of *B. pertussis* bacteria into the nares of BALB/c mice. In contrast, Wilk and colleagues performed an aerosol infection challenge and recovered bacteria by nasal wash, possibly not recovering the bacteria growing in a biofilm on the nasal septum [54] and thus obtained less than 10^3^ CFUs from the nasal cavities of challenged animals. At the high initial infection dose used by us, with homogenization of noses prior to plating, only a modest initial reduction of the nasal CFU load occurred in the wP-vaccinated animals, and a modest proliferation of bacteria was observed in the nasal cavity of sham-vaccinated animals over the first 7 days. The protective CD4^+^ T_RM_ response elicited by the wP vaccine [23,27,28], if induced, would appear as insufficient for full containment of a high infectious dose in the likely absence of specific sIgA immunity. Indeed, the vaccines were administered parenterally and even the wP vaccine most likely did not trigger any *B. pertussis*-specific sIgA response. Hence, the T_RM_-produced IL-17, which upregulates antimicrobial peptides and innate polyspecific sIgA in mucosal secretions and/or the IL-17-elicited attraction of neutrophils to the infected mucosa in wP-vaccinated animals, would only partially contain a high bacterial load. Nevertheless, we still believe that there is a real value to the use of the wP vaccine. In the natural situation of aerosol-transmitted infection, the *B. pertussis* dose received by a human is likely to be much lower than the dose used here for infection of mice that are quite resistant to *B. pertussis* colonization [55]. The spectacular control of pertussis incidence following the introduction of wP vaccine in the developed countries and the persisting control of pertussis in countries still using the wP vaccine clearly suggest that the wP vaccine not only prevents clinical pertussis, but also importantly reduces carriage and transmission of the pathogen.

Previous studies reported that addition of different protective antigens improved the efficacy of aP vaccines in the mouse lung infection clearance model and extended lung protection from *B. pertussis* infection. Queenan and coworkers reported improvement of a dPT plus FHA-comprising aP vaccine efficacy upon addition of FIM2/3 [52]. Addition of the adenylate cyclase toxoid or of the dACT-derived RTX antigen also improved the protective efficacy of a diluted aP vaccine in the mouse lung clearance model [36,37]. Indeed, we also show here that addition of FIM2/3 with *B. pertussis* LOS importantly improved the efficacy of the 1/20 HD of commercial aP vaccine-elicited immune protection against mouse lungs infection by *B. pertussis* (c.f. Figure 3a). Curiously, this was not further enhanced by addition of high amounts of the LPS-free dACT antigen (30 μg) that triggered ACT-neutralizing antibodies and hence was expected to enhance opsonophagocytic clearance of *B. pertussis* infection from mouse lungs. This is surprising, as *E. coli* LPS-contaminated dACT alone was previously found to be a potent protective antigen in the BALB/c mouse lung clearance model [33,34]. Moreover, an LPS-free dACT vaccine also conferred some protection from *B. pertussis* infection of mouse lungs [35]. It deserves to be explored why addition of LPS-free dACT resulted in somewhat reduced titers of antibodies against the other antigens (e.g., dPT, FHA and PRN, Figure S2) and thus blunted slightly the enhanced mouse lung protection from infection conferred by FIM2/3 and LOS addition into the diluted aP vaccine. It is possible that too high an amount of the immunodominant dACT antigen was used here.

Using an mScarlet-expressing fluorescent *B. pertussis* strain, we have developed an opsonophagocytic uptake assay for assessment of functional activity of antibodies induced by the aP vaccine and observed that in the absence of aP supplementation, the sera elicited by 1/20 HD of the aP vaccine (Infanrix^®^) performed poorly in the OPA assay. Serum from mice vaccinated with the 1/20 HD of aP vaccine supplemented with FIM2/3 and LOS mediated an enhanced opsonophagocytic uptake of *B. pertussis* by differentiated human HL-60 cells. Nevertheless, addition of FIM2/3 and LOS into the diluted aP vaccine did not confer any improved protection from nasal colonization by *B. pertussis*. Counterintuitively, addition of the dACT antigen into the diluted aP vaccine allowed the induction of ACT-neutralizing antibodies, but caused some inhibition of the capacity of the immune sera obtained to mediate opsonophagocytic uptake of *B. pertussis* bacteria. This was likely due to reduced titers of the opsonizing antibodies recognizing primarily the LOS, PRN and FIM2/3 antigens (c.f. Figure S3).

The cAMP-elevating action of ACT was previously shown to near-instantly ablate the bactericidal capacities of neutrophils, such as their oxidative burst and opsonophagocytic killing capacities [39,56,57]. ACT also compromises the capacity of neutrophils to phagocytose antibody-opsonized *B. pertussis* bacteria, and this inhibition can be alleviated by neutralization of ACT by antibodies of the immune sera [58,59]. Therefore, it is generally assumed that ACT-neutralizing antibodies might play an important role in fostering of bactericidal action of phagocytes in the initial stages of airway infection. However, the production of ACT-neutralizing serum IgG did not enhance the clearance of *B. pertussis* from the lungs and the nasal cavity in the experiments reported here. When dACT was added into the aP vaccine used, the neutralization of ACT activity appeared to have been negatively compensated by the reduction of titers of the opsonizing antibodies. Furthermore, induction of ACT-neutralizing secretory IgA on the airway mucosa may be needed for enhanced immune protection in the nose. Indeed, the major limitation of most of the reported mouse vaccination studies, including this one, is that the aP vaccines were applied parenterally and not mucosally. Hence, secretory IgA responses were not induced at the nasal mucosa. Humoral responses were, indeed, efficiently elicited upon nasal colonization by a live attenuated *B. pertussis* vaccine strain BPZE1 that triggered protection against subsequent nasal colonization by virulent *B. pertussis* [25].

Pertussis is a re-emerging infectious disease in many countries with high aP vaccine coverage. The licensed alum-adjuvanted aP vaccines efficiently prevent clinical pertussis disease in the first years after primovaccination. However, experimental evidence from a non-human primate model of infected olive baboons (*Papio Anubis*) would suggest that the aP vaccine does not prevent *B. pertussis* colonization of the respiratory tract and transmission to aP-vaccinated or unvaccinated animals [19]. Our data obtained in the mouse model are consistent with data from the baboon model and provide further evidence on the limitations of the current aP vaccines. The pertussis resurgence and outbreaks occurring in highly aP-vaccinated populations of many developed countries point to a high level of more-or-less asymptomatic and undiagnosed nasopharyngeal carriage and transmission of *B. pertussis* by aP-vaccinated individuals [18,20]. It will be important to determine what role in this phenomenon is played by the predominant Th2 polarization of aP-induced immune responses, absence of induction of mucosa-homing T_RM_ cells and the narrow spectrum of antigens delivered by the aP vaccine, respectively, as compared to the Th1-polarizing and broad antigenic spectrum delivered by wP vaccines. A plausible component of the aP vaccine problem could also be an “original antigenic sin” elicited by the chemically detoxified PT (dPT) toxoid. dPT has tertiary structure collapsed due to the chemical treatment that destroys about 80% of the conformational epitopes targeted by the toxin-neutralizing antibodies [60]. Plausibly, then, aP-vaccinated individuals respond to production of native PT in the course of infectious challenge primarily with non-neutralizing antibodies recognizing neoepitopes and linear epitopes of dPT, that may eventually hamper the neutralization of native PT action by a mechanism of linked epitope suppression. Indeed, Auderset and co-workers recently showed that booster vaccination with an experimental aP vaccine containing the genetically detoxified PT with preserved neutralizing epitopes and a TLR9A adjuvant, yielded a superior Th1-associated IgG2a antibody response capable to boost the aP prime, which translated into improved protection against a *B. pertussis* challenge [61].

Accumulated clinical evidence from the large-scale use of aP vaccine in humans clearly shows that levels of native PT-neutralizing antibodies elicited by the aP vaccine in primovaccinated infants are sufficient for conferring a life-saving protection against systemic effects of PT action and for prevention of clinical pertussis disease symptoms, such as hyperleukocytosis. However, given the parenteral application of the vaccine, the PT-neutralizing IgG antibody levels elicited by the present aP vaccine may be insufficient for controlling the local suppression of innate and adaptive immune defense mechanisms of the upper airway mucosa by the native PT released by the *B. pertussis* closely adhering to the ciliated epithelial cells of the nasopharynx. Hence, insufficient neutralization of the locally produced PT in mucosal tissue may support the establishment of long-term bacterial colonization. This hypothesis can now be tested with a reformulated aP vaccine containing the genetically detoxified gdPT antigen with superior protective immunogenicity, which has a preserved tertiary structure with fully preserved conformational neutralizing epitopes [62,63,64,65].

## 5. Conclusions

Immunization of BALB/c mice with a reduced dose of the aP vaccine ablated their natural capacity to eliminate *B. pertussis* infection from the nasal cavity and promoted persistent nasal colonization. In contrast, sham-vaccinated mice that received only the alum adjuvant in PBS were still able to clear the nasal infection as efficiently as the wP-vaccinated mice. This shows that it is not the adjuvant alone that compromises the innate capacity of mice to clear the nasal infection. Our results suggest that some component common to the two different formulations of the aP vaccines used (dPT or FHA, or both in synergy) prevents the mobilization of effective innate mucosal defense and inhibits the development of an adaptive mucosal immune response required for effective clearance of high-dose *B. pertussis* infection from the nose. It will be important to decipher the mechanism responsible, since extended nasopharyngeal colonization was previously observed also in aP-vaccinated and *B. pertussis*-challenged non-human primates [19], a model that mimics the natural infection of human upper airways by *B. pertussis* more faithfully than the mouse nasal clearance model used here. [62].

## Figures and Tables

**Table 1:** Composition of the used vaccines.

Vaccine	Brand Name	dPT (µg)	FHA (µg)	PRN (µg)	FIM2/3 (µg)	LOS (EU)	dACT (µg)	Al^3+^ (µg)
1/160 aP Hex	Hexacima^®^	0.156	0.156	-	-		-	137
1/160 aP Hex + FIM2/3 (1 µg)		0.156	0.156	-	1	6000	-	
1/160 aP Hex + FIM2/3 (3 µg)		0.156	0.156	-	3	18,000	-	
1/160 aP Hex + FIM2/3 (1 µg) + dACT		0.156	0.156	-	1	6000	30	
1/160 aP Hex + FIM2/3 (3 µg) + dACT		0.156	0.156	-	3	18,000	30	
1/20 aP Hex	Hexacima^®^	1.25	1.25	-	-		-	
1/20 aP Hex + FIM2/3 + PRN		1.25	1.25	0.2	5	30,000	-	153
1/20 aP Hex + FIM2/3 + PRN + dACT		1.25	1.25	0.2	5	30,000	30	
1/20 aP Inf	Infanrix^®^	1.25	1.25	0.4	-		-	
1/20 aP Inf + FIM2/3		1.25	1.25	0.4	5	30,000	-	131
1/20 aP Inf + FIM2/3 + dACT		1.25	1.25	0.4	5	30,000	30	
¼ wP Penta	Pentavac^®^ SD	nd	nd	nd	nd	nd	nd	≤312

dACT, detoxified adenylate cyclase toxin (188GS189); dPT, detoxified pertussis toxin; FHA, filamentous hemagglutinin; FIM2/3, fimbriae types 2 and 3; Hex, Hexacima^®^; Inf, Infanrix^®^; LOS, lipooligosaccharide; EU, endotoxin unit; Penta, Pentavac^®^; PRN, pertactin; nd—not determined.

## Supplementary Materials

The following are available online at https://www.mdpi.com/2076-393X/8/4/695/s1, Figure S1: Determination of the aP vaccine dose conferring a limited protection against intranasal *B. pertussis* challenge in BALB/c mice. Figure S2: Neutralization of toxin activities of ACT by sera of immunized mice. Figure S3: Total serum IgG antibody responses to the five vaccines antigens.

## Abbreviations

ACT	adenylate cyclase toxin
AG	antigens
aP	acellular pertussis
CFUs	colony forming units
dACT	adenylate cyclase toxoid
D-MEM	Dulbecco’s Modified Eagle’s Medium
dPT	detoxified pertussis toxin
DTwP	diphtheria–tetanus–whole-cell pertussis vaccine
EU	endotoxin unit
FHA	filamentous hemagglutinin
FIM	fimbriae
HD	human dose
LOS	outer membrane lipooligosaccharide
LPS	lipopolysaccharide
MOI	multiplicity of infection
OPA	opsonophagocytic uptake assay
PBS	phosphate-buffered saline
PMA	phorbol myristate acetate
PRN	pertactin
PT	pertussis toxin
PRMI	Roswell Park Memorial Institute medium
RTX	repeats in toxin
TdaP	tetanus–diphtheria–acellular pertussis vaccine
T_RM_	tissue resident memory T cells
wP	whole-cell pertussis
