# Supplementary material for: Acellular Pertussis Vaccine Inhibits Bordetella pertussis Clearance from the Nasal Mucosa of Mice
Source: Vaccines (Basel). 2020 Nov 19;8(4):695. doi: 10.3390/vaccines8040695 (PMC7711433; doi:10.3390/vaccines8040695)
Supplement: Supplementary file 1 [file vaccines-08-00695-s001.pdf]

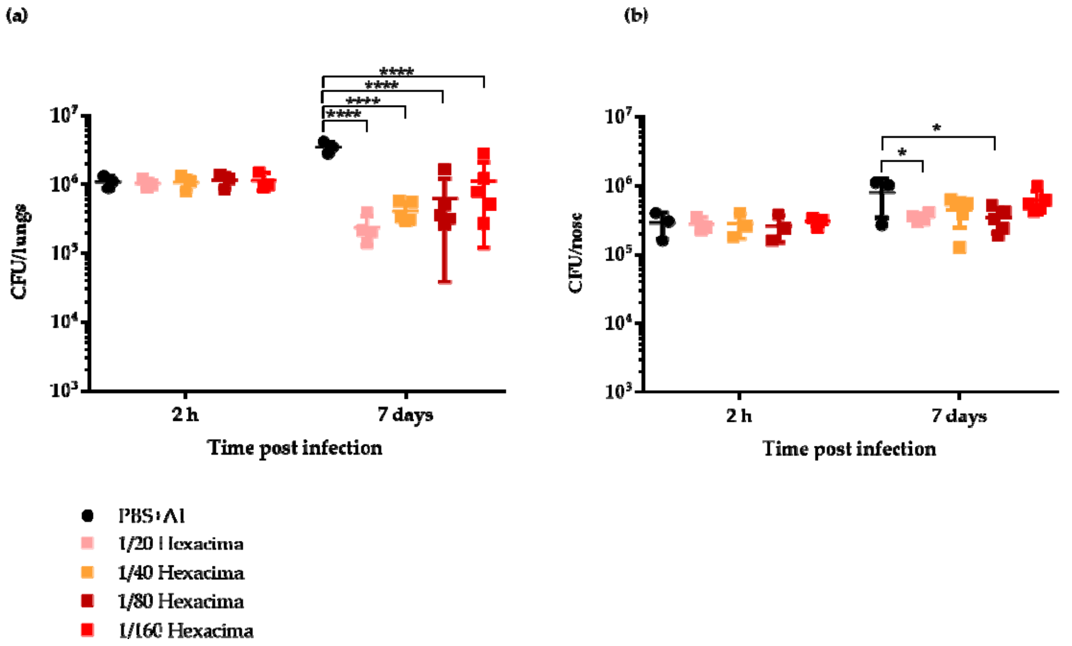

Figure S1. Determination of the aP vaccine dose conferring a limited protection against intranasal *B. pertussis* challenge in BALB/c mice. Mice were immunized twice intraperitoneally by injections on days 0 and 14 of different dilutions of the aP vaccine (1/20 HD Hexacima®, 1/40 HD Hexacima®, 1/80 HD Hexacima® and 1/160 HD Hexacima®) in PBS + Alum (0.208% w/V). Mice injected with PBS + Alum (0.208% w/V) alone served as negative controls. The animals were challenged intranasally with *B. pertussis* BPSM (8.6 x 10^5 CFU in 2 x 10 µl) on day 35. Mice were sampled at day 0 + 2 h and day 7 post challenge for enumeration of bacteria in the lungs (**a**) and in the nasal cavity (**b**). Results represent the bacterial counts for three to five mice per group ± SD. Two way ANOVA followed by Tukey´s multiple comparison test was used to analyze the statistical significance between groups. Only significant differences are indicated. * ($P < 0.05$); **** ($P < 0.0001$).

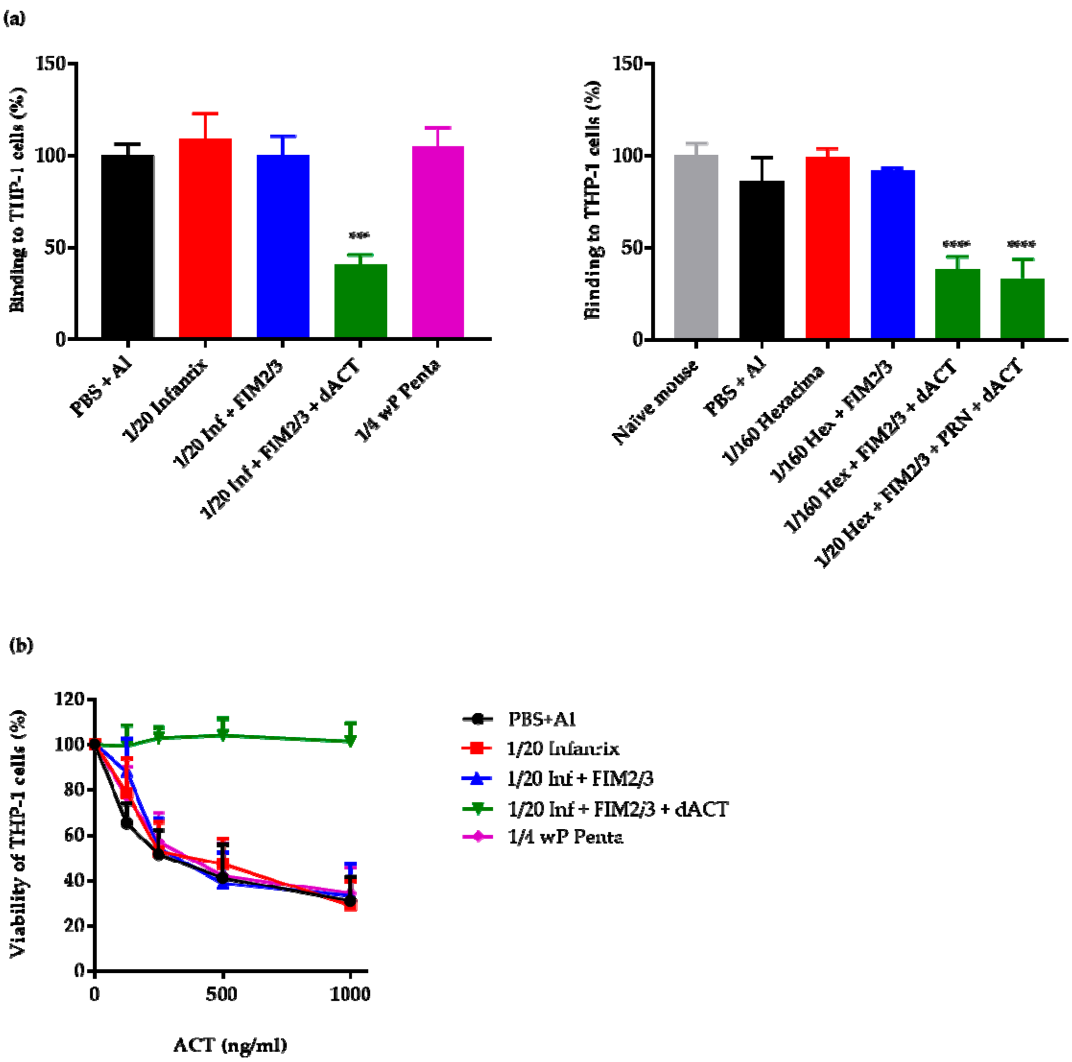

Figure S2. Neutralization of toxin activities of ACT by sera of immunized mice. **(a)** Inhibition of ACT binding to THP-1 monocytic cells by sera of immunized mice. ACT (1 µg/ml) was preincubated in D-MEM medium (without FCS) for 15 minutes on ice in the presence of the respective serum diluted 1:50 before THP-1 cells (10^6) were added and the mixture was incubated for additional 30 min at 4°C. Unbound ACT toxin was removed by three washes in D-MEM and after the transfer to a fresh tube, the cells were lyzed with 0.1% Triton X-100 for determination of cell-bound AC enzyme activity. ACT binding in the presence of naïve serum (PBS + Alum) was taken as 100%. N=4 biological replicates performed in duplicates. Data shown as mean ± SD. Only significant differences vs. negative control mice are indicated. *** ($P < 0{,}001$); **** ($P < 0.0001$). **(b)** Neutralization of ACT cytotoxic activity by sera of immunized mice. ACT (125-1,000 ng/ml) was incubated with serum samples (dilution 1:50) for 15 min at room temperature in D-MEM medium and the samples were then added to the THP-1 monocyte/macrophage cells (1.5 x 10^5). Cells were incubated at 37°C for 2 h in a humidified air/5% CO_2 atmosphere and the number of surviving cells was determined using the Cell proliferation reagent kit WST-1 (Roche Applied Science). The viability of control cells (buffer treated only) was taken as 100%. The % of viable cells represent average values from two different experiments performed in triplicates (N=2).

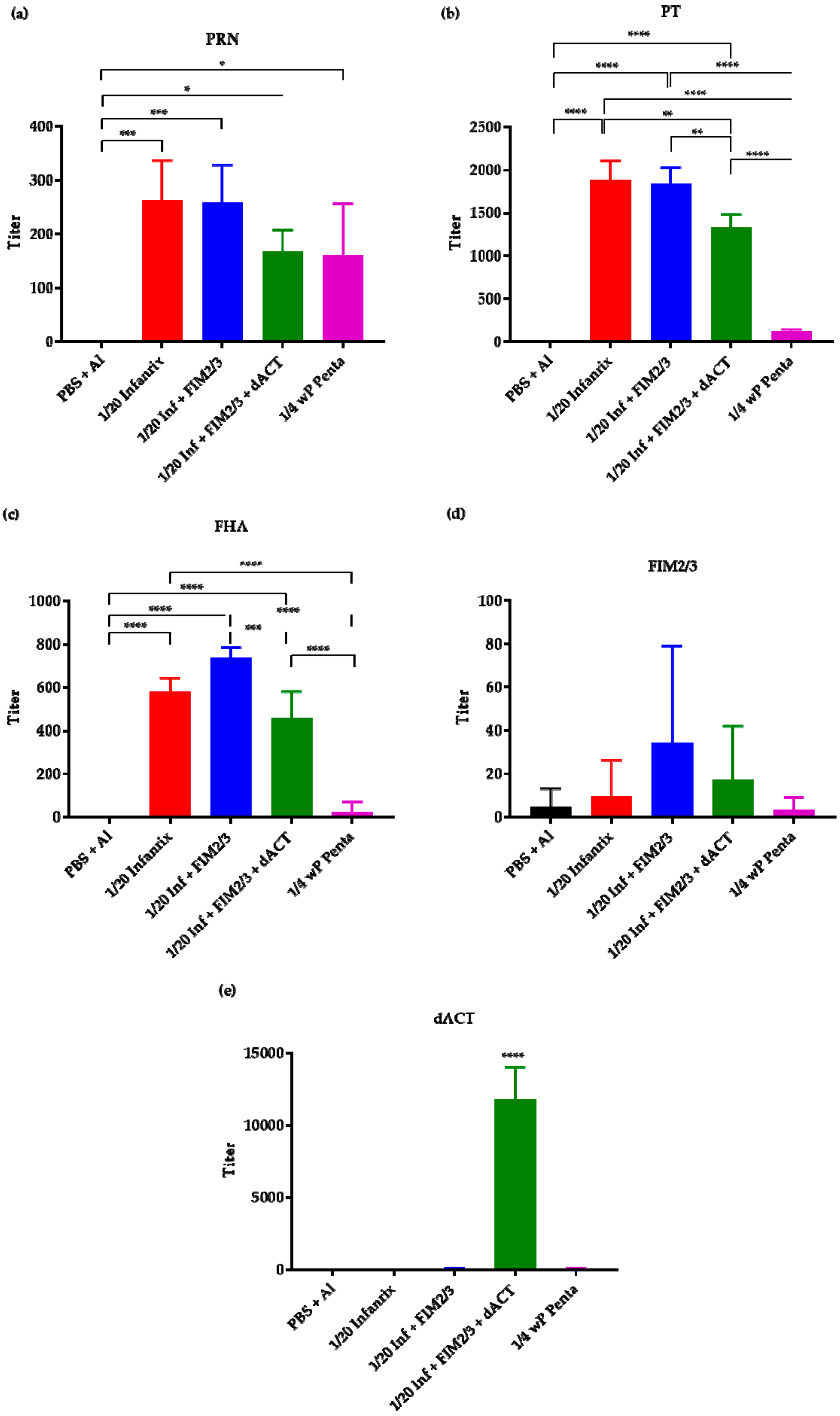
(a)
PRN
Titer
400
300
200
100
0
PBS + Al
1/20 Infanrix
1/20 Inf + FIM2/3
1/20 Inf + FIM2/3 + dACT
1/4 wP Penta
(b)
PT
Titer
2500
2000
1500
1000
500
0
PBS + Al
1/20 Infanrix
1/20 Inf + FIM2/3
1/20 Inf + FIM2/3 + dACT
1/4 wP Penta
(c)
FHA
Titer
1000
800
600
400
200
0
PBS + Al
1/20 Infanrix
1/20 Inf + FIM2/3
1/20 Inf + FIM2/3 + dACT
1/4 wP Penta
(d)
FIM2/3
Titer
100
80
60
40
20
0
PBS + Al
1/20 Infanrix
1/20 Inf + FIM2/3
1/20 Inf + FIM2/3 + dACT
1/4 wP Penta
(e)
dACT
Titer
15000
10000
5000
0
****
PBS + Al
1/20 Infanrix
1/20 Inf + FIM2/3
1/20 Inf + FIM2/3 + dACT
1/4 wP Penta

Figure S3. Total serum IgG antibody responses to the five vaccines antigens. **(a-e)** Serum IgG antibody responses to the five vaccines antigens – PRN, PT, FHA, FIM2/3 and dACT were measured by ELISA. Mice were immunized twice with the indicated vaccines and sera for antibody determinations were obtained on day 35. Results represent the mean antibody titers determined as the inflection points of the titration curves ± SD. Pool of sera of 6 mice per group were used in triplicates and two way ANOVA followed by Tukey´s multiple comparison test was performed to analyze the statistical significance between groups. * ($P < 0.05$); ** ($P < 0.01$); *** ($P <$ 0,001); **** ($P < 0.0001$).
